# A national strategy for a Canadian limb loss and limb difference registry

**DOI:** 10.33137/cpoj.v9i1.46909

**Published:** 2026-03-06

**Authors:** A.L Mayo, S.L Hitzig, D Zidarov, C MacKay, K.R Kaufman, V.K Noonan, J Andrysek, F Azhari, K.L Best, M Robert, S Dilkas, J Campbell, A Eshraghi, N Dudek, E.D Lemaire, S.J.T Guilcher, B Nowrouzi-Kia, N Habra, S Jean, J.S Hebert, S Dobrowolski, P.E Hoosein, M.W Payne, S King, H Underwood, B Pousett, J.O Totosy de Zepetnek, A Zucker-Levin, A Saporta, K Wong, S Glasford, S Hedlund, J Ellis, A Wong, B Yang, K Roddick, S.U Raschke, W.S Journeay, L Laakso, B Kok, B Moschiar Almeida, A Richardson, A Aternali, J Tiessen, W.C Miller

**Affiliations:** 1 St. John’s Rehab, Sunnybrook Health Sciences Centre, Toronto, Ontario, Canada.; 2 St. John’s Rehab Research Program, Sunnybrook Research Institute, Sunnybrook Health Sciences Centre, Toronto, Ontario, Canada.; 3 Temerty Faculty of Medicine, University of Toronto, Toronto, Ontario, Canada.; 4 Centre de recherche interdisciplinaire en réadaptation du Montréal métropolitain, Institut universitaire sur la réadaptation en déficience physique de Montréal, Centre intégré universitaire de santé et de services sociaux du Centre-Sud-de-l’Île-de-Montréal, Montréal, Québec, Canada.; 5 Université de Montréal, Montreal, Quebec, Canada.; 6 School of Rehabilitation Therapy, Queen's University, Kingston, Ontario, Canada.; 7 Mayo Clinic, Rochester, Minnesota, United States of America.; 8 Praxis Spinal Cord Institute, Vancouver, British Columbia, Canada.; 9 Bloorview Research Institute, Holland Bloorview Kids Rehabilitation Hospital, Toronto, Ontario, Canada.; 10 Institute of Biomedical Engineering, University of Toronto, Toronto, Ontario, Canada.; 11 Department Mechanical & Industrial Engineering, University of Toronto, Toronto, Ontario, Canada.; 12 Université Laval, Quebec City, Quebec, Canada.; 13 Centre interdisciplinaire de recherche en réadaptation et intégration sociale (Cirris), Centre intégré universitaire de santé et de services sociaux de la Capitale-Nationale (CIUSSS-CN), Quebec City, Quebec, Canada.; 14 West Park Healthcare Centre, University Health Network, Toronto, Ontario, Canada.; 15 Department of Medicine (Division of Physical Medicine & Rehabilitation) and The Ottawa Hospital, University of Ottawa, Ottawa, Ontario, Canada.; 16 Faculty of Medicine, University of Ottawa, Ottawa, Ontario, Canada.; 17 Leslie Dan Faculty of Pharmacy, University of Toronto, Toronto, Ontario, Canada.; 18 Centre intégré universitaire de santé et services sociaux du Centre-Sud-de-l’île-de-Montréal, Montreal, Quebec, Canada.; 19 University of Alberta, Edmonton, Alberta, Canada.; 20 Glenrose Rehabilitation Hospital, Edmonton, Alberta, Canada.; 21 Western University, London, Ontario, Canada.; 22 Parkwood Institute, London, Ontario, Canada.; 23 G.F. Strong Rehabilitation Centre, Vancouver, British Columbia, Canada.; 24 University of British Columbia, Vancouver, British Columbia, Canada.; 25 Rehabilitation Sciences, University of British Columbia, Vancouver, British Columbia, Canada.; 26 Centre for Aging SMART, Rehabilitation Research Program, G.F. Strong Rehabilitation Centre, Vancouver, British Columbia, Canada.; 27 Barber Prosthetics Clinic, Vancouver, British Columbia, Canada.; 28 Kinesiology and Health Studies, University of Regina, Regina, Saskatchewan, Canada.; 29 School of Rehabilitation Science, University of Saskatchewan, Saskatoon, Saskatchewan, Canada.; 30 Sunnybrook Centre for Independent Living, Sunnybrook Health Sciences Centre, Toronto, Ontario, Canada.; 31 Russel Prosthetics Ltd., New Westminster, British Columbia, Canada.; 32 Orthotics Prosthetics Canada, Canada.; 33 Dalhousie University, Saint John, New Brunswick, Canada.; 34 University of New Brunswick, Fredericton, New Brunswick, Canada.; 35 Stan Cassidy Centre for Rehabilitation, Fredericton, New Brunswick, Canada.; 36 British Columbia Institute of Technology, Vancouver, British Columbia, Canada.; 37 Dalhousie Medicine New Brunswick, Dalhousie University, Saint John, New Brunswick, Canada.; 38 The Moncton Hospital, Horizon Health Network, Moncton, New Brunswick, Canada.; 39 Custom Orthotic Design Group Ltd, Mississauga, Ontario, Canada.; 40 Winnipeg Prosthetics & Orthotics, Winnipeg, Manitoba, Canada.; 41 Schulich Heart Research Program, Sunnybrook Research Institute, Sunnybrook Health Sciences Centre, Toronto, Ontario, Canada; 42 George Brown College, Toronto, Ontario, Canada.; 43 Holland Bloorview Kids Rehabilitation Hospital, Toronto, Ontario, Canada.; 44 Department of Psychology, Faculty of Health, York University, Toronto, Ontario, Canada.; 45 Thrive Magazine, Grimsby, Ontario, Canada.; 46 Department of Occupational Science and Occupational Therapy, University of British Columbia, Vancouver, British Columbia, Canada.

**Keywords:** Amputation, Limb Difference, Registry, Consensus Workshop, Limb Loss, Rehabilitation, Canada

## Abstract

Canada lacks a national data source on individuals with limb loss and limb difference (LLD), which limits understanding of incidence, prevalence, risk factors, etiology, and healthcare outcomes. In the absence of standardized data collection, the provision of LLD healthcare services in Canada remains inconsistent. The objective of this study was to gather key interest groups’ perspectives on the development of a Canadian LLD registry. Invitees were identified through professional networks and snowball recruitment techniques. A two-round modified Delphi approach was utilized to identify key LLD registry domains via a pre-meeting survey followed by a virtual workshop on February 14, 2024. Of 96 invitees, 53 completed the survey, and 64 attended the workshop (63 from Canada and 1 from the United States). Five key LLD registry domains were identified: representation, standardization, practice-based evidence, research and innovation, and policy and funding. Inclusivity of diverse populations, national outcome measures adoption, and integration of psychosocial and clinical data was emphasized. Foreseen challenges included privacy concerns, necessary infrastructure, and resources to ensure long-term sustainability. Despite these challenges, a Canadian LLD registry could support advocacy, strengthen practice-based evidence, enhance research collaboration, improve clinical care, and inform population-level policies. Efforts to develop the registry are ongoing.

## INTRODUCTION

Amputation can result from trauma including motor vehicle accidents, work-related injuries, burns and military/combat incidents.^1,2^ Non-traumatic causes of amputation include dysvascular complications (e.g., diabetes and peripheral vascular disease), malignancy, frostbite, and septic shock.^3–5^ In addition, there are many causes for congenital limb differences (e.g., genetic factors, maternal exposures, disrupted blood flow).^6^ Individuals with an acquired traumatic and non-traumatic amputation, as well as individuals with congenital limb difference, experience significant mobility, functional, health and psychosocial challenges.^1,4,7–14^ International data suggest that amputations and limb differences have a significant economic impact.^15–18^ However, Canadian healthcare cost data are limited, restricting evidence-informed decisions for meeting patient needs and efficiently allocating resources.

While limb loss accounts for a significant portion of the “global burden of disability”,^3,19^ the disability-related impacts of limb loss and limb difference to Canada is unknown. Extrapolating amputation-related impacts from global data or other countries is unlikely to be accurate given differences in healthcare systems and funding, socio-economic conditions, geography (i.e., rural versus urban), exposure to war, conflict, and disease, climate, culture, and safety regulations. For example, in many parts of the world, trauma is the leading cause of amputation^19^ whereas in Canada, dysvascular amputations due to complications of diabetes and other factors (e.g., smoke exposure, hypercoagulable states) are the dominant cause and are increasing.^20^ As well, other countries that share predominance of dysvascular amputation, such as the United States (U.S.),^21^ vary greatly in healthcare funding and delivery models. Furthermore, there are significant differences within Canadian provinces as it relates to rehabilitation for people with limb loss and provision of prosthetic devices.^22,23^ The lack of data on the economic impact and variation in service delivery across Canada limits evidence-informed care and the effective allocation of resources for this patient population. A national registry could serve as an ideal mechanism to provide insights on who is living with an amputation or limb difference, what care is needed and has been provided, and the impact and outcomes of limb loss and limb difference (LLD) in the Canadian population.

Clinical registries are useful and widely used internationally across healthcare systems for understanding trends and outcomes in different patient cohorts. A clinical registry is defined as *‘an organized system that uses observational study methods to collect uniform data (clinical and other) to evaluate specified outcomes for a population defined by a particular disease, condition or exposure, which serves predetermined scientific, clinical or policy purpose(s)*’.^24^ The data collected by registries typically have standard definitions and have linkages to the original data sources to yield a centralized and comprehensive dataset.^24^ A registry also enables the organization of patient socio-demographic and clinical data, and stimulates and informs research.^25^ Registries can also provide real-world evidence concerning the impact of treatment and service delivery on outcomes.^25^ Importantly, they enable the comparison of care processes and patient outcomes across multiple sites.^26^ A systematic review found that 16 of 17 included papers reported improved healthcare processes and outcomes after implementing a registry.^25^ The review noted that registries can generate performance feedback for physicians, identify patients who are not receiving guideline-based care or who are at high risk and require additional monitoring, create reminders for patients, and reduce regional differences.^25^ Registries also provide an ideal platform for generating research questions and facilitating descriptive studies, clinical trials, and health services research.^25^

Limb loss registries have been successfully introduced in Sweden (SwedeAmp),^27^ Germany,^28^ and the U.S.^29,30^ For example, in response to gaps in data specific to the limb loss community in the U.S, the National Limb Loss and Preservation Registry (LLPR) was launched in 2020 using a quality improvement (QI) framework.^29,30^ To-date, there have been over 500,000 unique patients represented in the LLPR system, with almost 15 million episodes of care collected.^31^ A similar registry in Canada could be an essential and potentially cost-effective tool to improve patient care and guide health resource allocation by providing reliable, valid clinical data for Canadian and regional evidence-based decision-making.

Since 2019, ongoing efforts have fostered greater cohesion within the Canadian limb loss field by identifying key priorities, including recognition of the need for a registry.^32^ In addition, an environmental scan of rehabilitation care sites across Canada identified substantial variability in the delivery and provision of rehabilitation and prosthetic care.^33^ However, the scan identified that there is sufficient capacity for centres to participate in a national registry. Canadian rehabilitation registries such as the Ontario Stroke Registry,^34–36^ and the Rick Hansen Spinal Cord Injury Registry (RHSCIR)^37^ prove that such initiatives can be successful in Canada.

Centralizing data across healthcare settings across Canada by means of a LLD registry, would have similar benefits to ones developed in the U.S, Germany and Sweden.^27–30^ A Canadian LLD registry would strengthen the evidence base to inform practice and policy and better address the health and social (e.g., return-to-work outcomes, housing) needs of the LLD patient population. Despite the clear need, no Canadian LLD registry exists to date. This paper describes a consensus workshop that aimed to initiate a national strategy for developing a Canadian LLD registry.

## METHODOLOGY

To advance the development of a Canadian national LLD registry, a pre-workshop survey and virtual consensus workshop were conducted to gather insights from a nationwide cross-sectoral set of key interest group representatives. The goal was to develop a strategy for moving the registry forward. Key interest groups included, but were not limited to, healthcare providers (e.g., physiatrists, surgeons, prosthetists, orthotists, occupational therapists, physical therapists), decision-makers (e.g., professional organizations such as Orthotics Prosthetics Canada, healthcare managers), researchers, advocates (e.g., The War Amps, Amputee Coalition of Canada), and amputees. To structure the workshop, our team adopted a modified Delphi method^38^ to obtain structured feedback from LLD experts and professionals, regarding their perspectives on a registry. A Delphi method is a structured group communication method for obtaining expert opinion about complex problems through a series of questionnaires and controlled feedback.^38^ Similar to past initiatives,^32,39^ we modified the Delphi approach by first developing a pre-meeting survey (**Appendix A**) to anonymously gauge the attitudes and perspectives of invited consultation attendees regarding a limb loss registry. The results were then shared with participants to stimulate discussion during the virtual workshop (non-anonymous). However, the aim of the workshop was to brainstorm ideas for registry development, and purposefully did not aim to have a mechanism for consensus at the workshop since it was designed to be more exploratory in nature rather than definitive.

As per our Research Ethics Board (REB) policy at the Sunnybrook Health Sciences Centre, the Ethics Review Assessment Tool (ER-SAT) was completed and the project was determined to be a QI project exempted from REB review. Participation in the survey and workshop was voluntary. Participants were informed about the purpose of the LLD registry consensus survey and workshop, as well as the planned dissemination of results through journal publication. Survey responses were collected anonymously.

### Key Interest Group Identification

Since 2019, our Sunnybrook St. John’s Rehabilitation LLD research team has established Canadian wide-linkages across sectors from various initiatives and research collaborations.^32,40–42^ We relied on professional clinical and research networks, Canadian LLD peer support and advocacy groups, professional organizations, and snowball recruitment methods to identify potential workshop attendees. As well, our team carried out an environmental scan of Canadian limb loss rehabilitation centres prior to the virtual workshop to assess whether a sufficient number of sites could support a registry and to identify commonalities in patient populations, services provided, and outcome measures collected.^33^ Hence, we also invited participants from the environmental scan to attend the workshop. Through outreach and consultation with professional partners across Canada, we identified 96 potential attendees, including five team members (Mayo A.L, Hitzig S.L, Zidarov D, MacKay C, Miller W.C) who organized the workshop.

### Pre-Meeting Survey and Materials

The 96 potential attendees were provided an online survey asking for Likert-scale opinions regarding the most important contributions of a national registry, the feasibility of establishing a registry within the next 5 years, and potential barriers to its implementation. Information on key interest group representation was also collected to inform the organization of the meeting (e.g., small group discussion assignments; see below). The pre-meeting survey was delivered via email to the 96 potential participants. The pre-meeting survey was open for 1.5 months, from early December 2023 to mid-January 2024. Prior to the online virtual workshop, we provided confirmed attendees with a summary of the environmental scan undertaken by our team.^33^

### Meeting Structure and Processes

The meeting was held virtually on the Zoom™ Platform on February 14, 2024. To orient attendees to discussions on a national registry strategy, we provided an overview of its rationale and invited speakers to share insights. This included a presentation by a representative from the Amputee Coalition of Canada, a not-for-profit organization led by amputees (https://amputeecoalitioncanada.org/), to highlight community needs and share their vision for a national registry. This was followed by presentations by Dr. Kenton R. Kaufman from the Mayo Clinic on the development of the U.S. LLPR, and Dr. Vanessa Noonan from the Praxis Spinal Cord Institute who presented on RHSCIR. In addition, one of our workshop organizing leads, Dr. Diana Zidarov from the Institut universitaire sur la réadaptation en déficience physique de Montréal and Université de Montréal, presented her research project, “*Development of a Pan-Canadian Core Outcome Set for Rehabilitation Management of Individuals with Lower Limb Amputation*,” and shared preliminary, unpublished findings. As well, Dr. Zidarov discussed efforts to inform core outcome data sets in lower limb loss through systematic reviews^43^ and the use of patient-oriented surveys to identify domains important to amputees and care providers.

The virtual workshop used a mix of small and large group discussions. The large group discussions (all 64 attendees) were facilitated by an independent neutral facilitator. The small group discussions (6-7 individuals per group were facilitated by one dedicated research staff member). Prior to the workshop, the core study team pre-assigned attendees to one of the small groups. Efforts were undertaken to have diversity in the small groups in terms of geographic location and key interest group representation. The Mural+App™ platform^44^ was used to structure the discussions and capture key ideas, which was managed by research meeting team members. The topics explored across the meeting included:

**1) Metrics for success:** What does a successful LLD registry look like to you? What is your vision for the registry? How can a registry lead to improved health and well-being (e.g., quality of life, community participation, mental health) for the LLD community?
**2) Enablers and Barriers:** What do you think will make it easy to develop and implement a registry? What will make it a challenge?
**3) Resources:** Who are the people/groups that should be involved in the planning and managing the registry? What are some ideas on how to obtain funding, resources, and expertise to move a strategy forward?

After each small group discussion, there was a large group report-back with opportunities for feedback. The neutral facilitator worked to consolidate key themes to inform a proposed strategy for the registry.

## RESULTS

Of the identified potential 96 attendees, 53 responded to the pre-meeting survey, and 64 attended the virtual workshop. Of the survey respondents, 21 were researchers; 14 were certified prosthetists or orthotists (9 prosthetists, 5 orthotists); 13 were physicians (limb loss physiatrists or surgeons); 7 were physical therapists; 6 were individuals with limb loss; 5 were decision-makers; 5 were community advocates; 4 were prosthetic engineers; 1 was a podiatrist; and 1 was an occupational therapist. Respondents could select multiple roles.

Of the 53 survey respondents, 30 individuals (57%) felt establishing a registry within the next 5 years (with at least three sites or more with a common dataset) was highly feasible, and 23 (43%) felt it was somewhat feasible. None reported it was not feasible. **Table 1** and **Table 2** details the contributions respondents envisioned a registry would make to the field, and perceived barriers to implementation, respectively.

**Table 1:** Survey respondents’ views on what they feel is the most important contribution a national registry would make to the field (N=53). Respondents could only select one option.

Response	Frequency	Percentage
Facilitating the collecting of standardized data and promoting data sharing	17	32.1%
Understanding trends & outcomes in different patient cohorts	9	17.0%
Providing real-world evidence of the effects of treatment & service delivery models on patient outcomes	8	15.1%
Supporting advocacy efforts	7	13.2%
Accelerating research (e.g. facilitating clinical trials, recruitment, etc.)	6	11.3%
Other important features: Creating a network Allow for refinement of curriculum I think these are all important and could be accomplished by various subgroups Provide concrete evidence for advancing support & care for individuals with limb loss, nationally and provincially. Liaison with OPC (Orthotics Prosthetics Canada)	5	9.4%
No response	1	1.9%

**Table 2:** Survey respondents’ views on barriers or challenges in establishing a Canadian registry (N=53). Respondents could select all that apply.

Response	Frequency	Percentage
Lack of funding	41	77.4%
Fears of additional workload burden to front line clinical staff	29	54.7%
Lack of connections across sectors (e.g. clinical, research, advocacy)	24	45.3%
Lack of consensus on a core set of outcome domains or outcome measures	23	43.4%
Lack of sufficient infrastructure across Canadian sites	20	37.7%
Privacy legislation related to personal health information	19	35.8%
Other Barriers (open-ended responses): The ability to collect a comprehensive and full set of data which would result in an incomplete picture making the data less robust. Ability to collect from acute care and private (prosthetic) sectors. Language (French); Coding of data (i.e., standardizing for data entry)	2	3.7%

### Online Workshop

At the online workshop, 64 attendees participated, all from Canada except one from the United States. The 63 Canadians were from Ontario (n=34), Quebec (n=10), British Columbia (n=8), Alberta (n=4), Saskatchewan (n=2), Manitoba (n=2), Nova Scotia (n=2), and New Brunswick (n=1). Eight discussion groups, including individuals with LLD, clinicians, researchers, policy representatives, and community organizations, provided perspectives on the development of a Canadian registry. From the small group discussions, five overarching domains emerged as being essential to registry development:

1. Representation,
2. Standardization,
3. Practice-based evidence,
4. Research and innovation,
5. Policy and funding.

Each domain captured both opportunities and barriers for establishing a sustainable, nationally relevant registry.

#### 1. Representation

Participants emphasized the importance of building a registry that reflects the diversity of Canadians with limb loss, including congenital differences, traumatic and non-traumatic amputations, and individuals with limb loss that are not prosthetic users. Ensuring inclusivity across regions, cultures, and health systems was seen as critical to achieving a representative dataset. Indigenous and marginalized communities were identified as priority populations, given disproportionate impacts of limb loss and barriers to care across Canada.

A key consideration was that developing a registry would require buy-in from key interest groups across sectors (**Table 3**), and that no single group should act as the gatekeeper to access. Meaning, that the registry should be organized so that it enables collaboration to answer key questions pertinent to the LLD community. One step to foster collective buy-in is to create a value statement that reflects the priorities of different sectors for a registry (i.e., a “What’s in it for me” strategy), ensuring the registry is perceived as valuable by all key interest groups. For example, this could involve engaging prosthetic and orthotic representatives from local, provincial, and national associations, as well as groups in the limb preservation field (e.g., organizations serving the diabetes, cardiac, and wound care communities). It would be important to reach out and engage with these organizations to see if there are existing datasets that could be leveraged to move a limb loss and limb difference registry forward.

**Table 3:** Key interest groups identified for registry development and management.

Key interest group	Examples cited by attendees
Individuals with lived experience	People with limb loss, congenital differences
Health professionals	Physiatrists, orthopedics, vascular surgeons, podiatrists, physiotherapists, caregivers
Non-profit organizations	The War Amps, Amputee Coalition of Canada, ISPO
Researchers	Universities, graduate trainees
Policy/funders	Ministries of Health, CIHI, hospital foundations
Technology/IT experts	API/EMR integration specialists, AI/data scientists

**AI** = Artificial Intelligence; **API** = Application Programming Interface; **CIHI** = Canadian Institute of Health Information; **EMR** = Electronic Medical Record; **ISPO** = International Society of Prosthetics and Orthotics.

Critically, there was agreement that persons with LLD should be engaged to ensure that the resulting registry would reflect their needs and priorities. Regardless, the engagement of different sectors will require a clear communication strategy to raise awareness about the importance of a registry, and to build accompanying mechanisms for additional feedback on its development.

#### 2. Standardization

While specific data elements and outcome measures were not fully discussed at the workshop, attendees underscored the need for national definitions (e.g., traumatic vs. non-traumatic, congenital limb differences) and standardized outcome measures, such as quality of life indicators. In addition to capturing data regarding acquired limb loss, there was an expressed desire to also capture data on congenital limb difference. The general consensus was that the registry data should be limited to items that could be reliably collected, and easily compared across participating sites. While a national approach was considered the ultimate goal, many suggested starting with provincial or single-centre pilots to establish proof of concept and to focus on specific groups of patients common to most Canadian centres (e.g., acquired transtibial amputations). Data collection challenges included inter-provincial healthcare differences, legal barriers to data-sharing, and variable use of electronic medical systems.

#### 3. Practice Based Evidence

Groups repeatedly emphasized that the registry should support a shift from “evidence-based practice” to “practice-based evidence.” Practice-based evidence is the use of real-world data collected from routine clinical care (e.g., outcome measures, patient feedback) to understand what works for a specific group of patients.^45^ Hence, it represents a continuous QI approach that offers direct feedback on ways to improve care in real time, under real clinical circumstances, with real patients and providers.^46^ When reviewing the differences between RHSCIR and the LLPR, the QI framework of the U.S. registry was highly appealing to the majority of meeting attendees. All attendees agreed that the ultimate goal of any registry would be to advance standards in care for the LLD community by showcasing where potential inequities may exist across the country. A research based model, such as RHSCIR, might be more limited in its ability to obtain large swaths of data since all RHSCIR participants undergo an informed consent process to allow for extended data collection on outcomes. Conversely, the LLPR’s QI framework uses routinely collected health data that is available in hospital’s electronic health records systems. This reduces data collection time and costs, enhances data quality, and supports scalability.

Beyond clinical outcomes, the registry was envisioned as a resource to capture psychosocial dimensions of LLD, including isolation, stigma, peer support, and mental health impacts as part of long-term clinical follow-up. This would allow clinicians and policymakers to better identify best practices and guide resource allocation using real-world data.

#### 4. Research and Innovation

Despite consensus on adopting a QI framework for a national registry, attendees agreed that it could also facilitate national collaborations in LLD research. As described, inequities in care identified through registry reports could guide in-depth research to better understand contributing factors. By enabling secondary data analysis of outcomes, research may be able to leverage these findings to innovate new care approaches for the LLD community. Additionally, researchers have access to sources of funding (e.g., national tri-council agency competitions) that could serve to expand on the registry or to fund projects that build on identified registry trends and population needs. Hence, there was a clear recognition that the research community can play a critical role in helping to optimize registry outcomes.

Additionally, some innovations were identified to facilitate registry data collection and management, including integration with electronic medical records and application programming interfaces (APIs) to reduce manual entry, as well as enabling patient-uploaded health data (e.g., online surveys). Identified opportunities enabled by a registry included exploring the use of artificial intelligence for predictive modeling of health trajectories, and linking with existing registries (e.g., spinal cord injury, stroke) to maximize efficiency and comparability. As noted above in the ‘Representation’ section, it will be critical to address the privacy and legal considerations on the collection, storage, and use of registry data.

#### 5. Policy and Funding

Funding and governance were identified as the most critical challenges. Participants noted that research grants could support start-up costs but would not ensure long-term sustainability of the registry. Models cited included RHSCIR, which benefited from strong champions and diversified funding sources. Suggested strategies included engaging government ministries, hospital foundations, philanthropic donors, and advocacy organizations. Many stressed the importance of having a visible, trusted champion to mobilize resources and public support.

Policy regarding the registry and the use of its data was not discussed in depth but it was stated that the registry should become established enough to influence policy and improve healthcare outcomes for amputees. Related to the discussion on ‘practice based evidence’ and ‘research and innovation’, the attendees discussed the importance of being able to leverage the data from the registry to influence policy decisions regarding the allocation of resources to meet the health and social care needs (e.g., return to work, housing, transportation) of people with LLD. As such, incorporating a strategy on how to use the registry to advocate for this population was seen as central to optimize its impact. At the same time, sustaining the registry will require dedicated funding, and highlighting its outcomes may help identify and attract potential long-term support from government, industry, and other key interest groups.

### Challenges and Solutions

Potential roadblocks and solutions to establishing a registry were identified. Funding was a prominent issue, starting with the funding needed to build a prototype. This included avoiding reliance on volunteers to establish and manage the registry, instead securing initial investments to support dedicated staff to run and mobilize it. Hospital foundations, non-profit organizations, industry, and research grants were identified as potential sources of initial investment. While some discussions addressed the start-up organization of a registry group (e.g., terms of reference, board structure and composition), attendees agreed that identifying a champion to lead these efforts was essential. Some national groups capable of collectively championing this initiative included the War Amps, Orthotics Prosthetics Canada (OPC), the Amputee Coalition of Canada, and the International Society of Prosthetics and Orthotics (ISPO) Canada. The attendees recognized the need for dedicated organizations to develop a strategy, and to communicate with the broader community to start developing and communicating a strategy to build buy-in across the country, which could lead to funding opportunities through lobbying efforts to different potential funders (e.g., federal government, advocacy groups, unrestricted industry grants).

Another challenge was around data ownership, data collection and data access. While the vision for the registry is to foster collaborations and to be inclusive, legal, ethical, and privacy considerations must be addressed. There was also strong emphasis on how data are collected, stored, and accessed. Registry experts from the already successful RHSCIR and LLPR can inform the development process, and provide insights on potential barriers and facilitators to setting up a Canadian registry. As described above, there was consensus that adopting a QI framework similar to the one used by LLPR, and starting with limited sites and data elements can potentially be the ideal approach to determining what data is feasible to collect. This exercise will be particularly critical for testing what could be extracted from existing electronic medical health records at different care sites (i.e., hospitals) across different provinces and territories.

## DISCUSSION

The aim of this virtual workshop was to develop a strategy for establishing a national LLD registry. Five interrelated domains were identified as being foundational to registration development: representations, standardization, practice-based evidence, research and innovation, and policy and funding. Representation emphasized cross sector contribution, including but not limited to individuals with lived experiences and marginalized communities. Standardization captured the need for national comparable definitions and core outcome measures. Practice-based evidence captured the preference for a QI-based registry to generate real-world data. Research and innovation emphasized national research collaboration supported by appropriate infrastructure, while policy and funding addressed sustainability through governance and diversified funding. Taken together, these domains suggest that national registry development requires coordinated attention to data infrastructure, alignment with real-world clinical practice, and sustainable governance and funding.

There was broad consensus that for a registry to be feasible within the next 5 years, efforts must prioritize a manageable, user-friendly design that is accessible to healthcare workers and researchers across the country. Critically, there is a need to identify dedicated sources of funding to launch a pilot of the registry, and to develop plans for long-term sustainability. If funding can be secured, it was agreed that starting with a limited scope, such as piloting the registry at the provincial level initially, enrolling only new patients, or initially focusing on specific patients (e.g., transtibial amputations), was a way to increase feasibility before expanding over time. Other registries have similarly started off small in scope and grew over time.^37,47^ For instance, RHSCIR opted to enroll only new injuries (acute cases) given the challenges of retrospectively mining data of patients with chronic SCI.^37^ Going forward, importance was placed on engaging a broad range of different Canadian communities and organizations, including rehabilitation facilities, as well as non-profit and advocacy agencies that can serve as potential collaborators or funders of the registry.

The implementation, maintenance and operation of a registry requires substantial effort,^48^ which makes them an expensive endeavor.^37^ It has been recommended that when defining the purpose of a registry, a balance is needed between the work required to achieve its objectives and the resources available.^49^ Unstable funding is a critical threat to registries.^50^ As such, the proposed strategy of starting a pilot registry that is small and relatively focused will provide critical insights on the resources needed to enroll participants, and to extract data in order to provide initial evidence on the feasibility and associated costs of a national registry. In addition to funding, several other issues require further consideration and planning (e.g., technological, legal).^48^ Importantly, the insights generated by analyzing data from registries make it possible to design tailored interventions.^24,51^ While motivation among key interest groups (e.g., care providers) has been cited as a challenge to registry sustainability,^47^ our team’s prior work identifying national priorities and conducting a preliminary environmental scan,^32,33^ together with the expressed interest of virtual workshop attendees, demonstrates strong support within the LLD community for the development and implementation of a national registry. These issues will need to be further discussed and mapped out as a strategy for a national registry unfolds.

Importantly, there are a number of registries managed by patient advocacy groups that operate similarly to registries led by research or clinical institutions except they are managed by patients and advocacy groups that are called ‘patient-powered registries’.^52^ The ownership of and access to data stored within the registry is a critical issue requiring further reflection since one of the intended goals of a national registry identified at the virtual workshop was to use it to strengthen advocacy efforts by mobilizing its epidemiological data to change clinical and community practice. As noted, there is a substantial lack of epidemiological data on LLD populations in Canada, and a registry could help fill these critical gaps, thereby better enabling key interest groups to advocate for health and social care resources. For example, a recent retrospective analysis of administrative health data for Ontarians with dysvascular lower limb loss (N = 10,905) found that patients with fewer comorbidities and better continuity of care had a lower risk of acute hospitalizations and emergency department visits.^53^ In this case, these data could be mobilized to advocate for additional resources to improve transitions from acute settings back to those in the community with elevated risk profiles. Given that the data from this study were Ontario specific, this limits the generalizability of these findings to others living across Canada. As such, a unified registry could contribute to large sample sizes to run more sophisticated analyses that can demonstrate national impact, while also showcasing where there may be disparities in care, and thereby provide the requisite evidence to better advocate for this population. Overall, registries offer several well-documented benefits, including providing epidemiological data (incidence, prevalence, mortality), supporting disease prevention and treatment monitoring, and informing healthcare planning.^24^ These functions could address critical questions in the field and contribute to significant advancements in care for this population.

It should be noted that the identified recommendations for the strategy were derived at a virtual workshop, which followed a Modified Delphi structure, but that no formal mechanism for achieving consensus was applied. The use of the Delphi method was employed to provide some structure for discussing the strategy, although given that the primary objective was exploratory in nature, consensus was not felt necessary. Specifically, our team avoided imposing constraints on brainstorming registry ideas, which might have occurred if consensus had been sought, as the goal was not to define the registry’s specific elements but to deliberate on a strategy to initiate its development. Considerable work remains to solidify a strategy, including identifying potential data elements (e.g., socio-demographics, impairment, prosthetic fitting) for a national registry and engaging key partners to support its long-term funding.

The issue of data elements were not fully discussed, and any group moving a strategy forward should review existing datasets, including those being used by the LLPR, ISPO’s Consensus Outcome Measures for Prosthetic and Amputation Services,^54^ and other available systematic reviews detailing outcomes for amputees.^43,55,56^ For example, the data elements in the LLPR were selected through a landscape analysis that identified valid administrative and clinical data, resulting in three types of elements:^29^

- **Type 1 – Hospital-level data:** patient information, amputation characteristics, and facility, provider, and payer details.
- **Type 2 – Provider/Prosthetic fitting data.**
- **Type 3 – Patient-reported outcomes:** including the Patient-Reported Outcomes Measurement Information System (PROMIS) Physical Function,^57^ Expanded Socket Comfort Score, and Activity-Specific Balance Confidence Scale.^58^

Registries such as these can serve as useful points of reference in the development of a Canadian registry. It should be noted however that the LLPR shares a common electronic health record system with an agreed-upon set of outcome measures, which is not the case in Canada.

Critically, there is a need to do additional in-depth consultation with the LLD community on the co-production/selection of data elements to ensure that the collected data is meaningful to them. Other registries, such as RHSCIR and the LLPR, have engaged a diverse set of key interest groups;^30,37^ which contributed to their successful start-up. Overall, the processes used to develop these registries (such as recruitment and data collection, data security, quality assurance, and governance), along with the key lessons learned, provide valuable insights to inform the development of a Canadian LLD registry strategy.

### Limitations

In terms of limitations, this workshop involved 64 professionals, primarily from the healthcare and research sectors, with only a small number of individuals with lived experience (amputees), and limited representation from marginalized or Indigenous communities. As well, the meeting was held on Zoom, and was only accessible to those who spoke English. This lack of diverse perspectives might impact the comprehensiveness of the recommendations. Although a modified Delphi approach was used, the workshop did not employ a formal mechanism for achieving consensus. As noted above, the process was exploratory rather than definitive, which may limit the strength and clarity of the recommendations.^1^ As well, this paper does not specify which data elements should be included in the registry, nor does it provide a detailed plan for data collection, management, or governance. There is a recognized need for further work to determine meaningful data elements and to ensure the registry reflects the needs of the LLD community in Canada. However, this summary of this event is not meant to be a definitive or rigid set of guidelines for establishing a national registry, but rather serves as a starting point to support the ongoing national dialogue on building capacity in the Canadian LLD field.^32^

## CONCLUSION

A virtual consensus workshop informed by a modified Delphi approach identified five foundational domains to guide the development of a Canadian national LLD registry, which include: representation, standardization, practice-based evidence, research and innovation, and policy and funding. These domains provide a proposed, key interest group–informed strategy for feasible and inclusive registry development, highlighting the need to address barriers such as data infrastructure, clinical alignment, and sustainable governance and funding. Such a LLD registry in Canada would strengthen advocacy efforts, the commitment to generating practice-based evidence within and beyond the healthcare system, while also fostering national research collaborations. Most importantly, a Canadian LLD registry would provide essential data that can be leveraged to improve and ensure more equitable LLD care across the country.

## Figures and Tables

**Figure:**
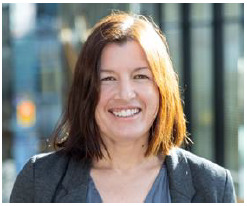


**Figure:**
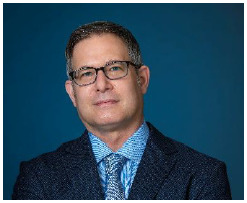


**Figure:**
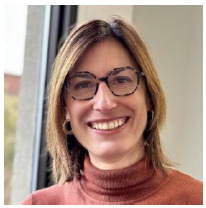


**Figure:**
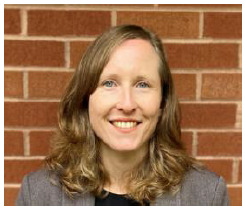


**Figure:**
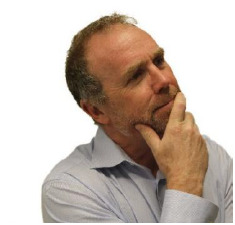


## AUTHORS BIOGRAPHY*

**Dr. Amanda Mayo**,** MD, MHSc, FRCPC, is a Physiatrist and Chief of Rehabilitation at Sunnybrook Health Sciences Centre and Associate Professor in the Department of Medicine at the University of Toronto. She subspecializes in Amputee Rehabilitation. Dr. Mayo is an Associate Director of the University of Toronto Centre for Quality Improvement and Patient Safety. Her research focuses on improving the continuum of care for individuals with limb loss, and digital fabrication of prosthetic and orthotic devices.

**Dr. Sander Hitzig** holds the John C. and Sally Horsfall Eaton Chair in Rehabilitation Research and is the Program Research Director and a Senior Scientist at the St. John’s Rehab Research Program at the Sunnybrook Research Institute. He is also an Associate Professor (Affiliated Scientist) in the Department of Occupational Science and Occupational Therapy and the Rehabilitation Sciences Institute (Temerty Faculty of Medicine) and in the Dalla Lana School of Public Health at the University of Toronto. He is also a board member on the International Society of Prosthetics and Orthotics (ISPO) Canada. Dr. Hitzig’s research program focuses on the long-term outcomes of people aging with a disability, and co-designs new models of care to optimize their health and wellbeing across the continuum of care.

**Dr. Diana Zidarov** is a research scientist at the Institut universitaire sur la réadaptation en déficience physique de Montréal and an adjunct professor at the School of Rehabilitation of the University of Montreal. She has extensive clinical experience as a physiotherapist working with individuals with upper and lower limb amputation. Her current research focuses on the development, implementation, and evaluation of novel interventions, including physical activity programs for individuals with limb loss living in the community. She is also interested in evaluating the quality of care in amputee rehabilitation programs, particularly through the development and implementation of quality indicators and clinical information systems. Dr. Zidarov collaborates closely with clinicians, patients, and organizations to strengthen care pathways and promote patient-centered approaches, including the use of meaningful outcome measures.

**Crystal MacKay** is a health services researcher with a clinical background in physical therapy. She is an Assistant Professor in the School of Rehabilitation Therapy, Queen’s Health Sciences at Queen’s University, an Affiliate Scientist at KITE Research institute, University Health Network and has a status appointment as an Assistant Professor in the Department of Physical Therapy, Temerty Faculty of Medicine, University of Toronto. She is a Member of Connected Minds, a collaborative research initiative at Queen’s and York Universities and a Board Member on the International Society of Prosthetics and Orthotics Canada. Her research aims to improve the quality and accessibility of health care for people living with disability or chronic conditions, such as limb loss.

**William C Miller (Bill),** PhD, FCAOT, an occupational therapist/epidemiologist, is a Professor in the Department of Occupational Science and Occupational Therapy at the University of British Columbia. He is a member of the International Society of Prosthetics Scientific Program Committee for the 2025 / 2027 World Congress’. Bill is a member of AGEWELL a pan-Canadian Network designed to create real-world assistive technology solutions that have a meaningful impact on the lives of Canadian seniors, their caregivers and individuals with physical disability. His research focuses on the epidemiology, measurement, and evaluation of interventions and technologies that are designed to remediate mobility disability with the goal of improving social participation. Recently, Bill has focused his work on digital technologies designed to improve the quality of life of individuals with lower limb amputation. He has developed 6 outcome tools designed for clinical practice and research which have been translated into multiple languages.

* Biographies were only provided for the corresponding author and workshop organizers.

** Corresponding author.

## APPENDIX

### APPENDIX A: PRE-MEETING SURVEY

The aim of the survey is to help inform the discussions for the planning meeting on a national limb loss registry.

**1. I am: (please select all that apply):**

□ A person with lower limb loss

□ A person with upper limb loss

□ A prosthetist

□ An orthotist

□ A P&O assistant

□ A physiatrist

□ A psychiatrist

□ A surgeon

□ A vascular surgeon

□ A trauma surgeon

□ Other MD (specify): ____________________

□ A physical therapist

□ A social worker

□ An occupational therapist

□ A PT/OT assistant

□ A psychologist

□ Other allied healthcare professional (please specify): ____________________

□ A registered nurse

□ A registered practical nurse

□ Other nursing specialty (please specify): ____________________

□ A decision-maker (e.g., hospital administrator, manager, etc.)

□ A policy-maker (e.g., municipal, provincial, federal; please specify): ____________________

□ A community advocate

□ Engineer

□ A university professor or research scientist

□ Other (please specify): ____________________

**2. I live/work in: (please select all that apply):**

□ Canada (please list province or territory): _________________________

□ United States (please list state): ________________________________

□ Other (please list): ___________________________________________

**3. What do you feel would be the most important contribution a national limb loss registry would make to the field? Is this a Select one (if so may want to put in brackets)**

□ Understanding trends and outcomes in different patient cohorts

□ Accelerating research (e.g., facilitating clinical trials, recruitment, etc.).

□ Providing real-world evidence of the effects of treatment and service delivery models on patient outcomes

□ Facilitating the collection of standardized data and promoting data sharing

□ Supporting advocacy efforts

□ Other (please describe): ____________________________________

**4. I believe that establishing an initial Canadian limb loss registry (at least 3 sites or more with a common dataset) within the next 5 years is:**

□ Highly feasible

□ Somewhat feasible

□ Not at all feasible

Please explain why? _________________________________________________

_________________________________________________________________________

_________________________________________________________________________

**5. What do you think are barriers or challenges in establishing a Canadian limb loss registry? Please select all that apply.**

□ Lack of funding

□ Lack of connections across sectors (e.g., clinical, research, advocacy)

□ Privacy legislation related to personal health information

□ Lack of sufficient infrastructure across Canadian sites

□ Lack of consensus on a core set of outcome domains or outcome measures

□ Fears of additional workload burden to frontline clinical staff

□ Other barrier(s) – please specify: ______________________________________________

**6. Please share any thoughts about the possibility of establishing a Canadian limb loss registry?**

_________________________________________________________________________

_________________________________________________________________________

**THANK YOU!**
